# Role of long-chain polyunsaturated fatty acids, eicosapentaenoic and docosahexaenoic, in the regulation of gene expression during the development of obesity: a systematic review

**DOI:** 10.3389/fnut.2023.1288804

**Published:** 2023-11-09

**Authors:** Cristian Sandoval, Karen Nahuelqueo, Luciana Mella, Blanca Recabarren, Vanessa Souza-Mello, Jorge Farías

**Affiliations:** ^1^Escuela de Tecnología Médica, Facultad de Salud, Universidad Santo Tomás, Osorno, Chile; ^2^Departamento de Ingeniería Química, Facultad de Ingeniería y Ciencias, Universidad de La Frontera, Temuco, Chile; ^3^Departamento de Ciencias Preclínicas, Facultad de Medicina, Universidad de La Frontera, Temuco, Chile; ^4^Carrera de Tecnología Médica, Facultad de Medicina, Universidad de La Frontera, Temuco, Chile; ^5^Laboratorio de Morfometría, Metabolismo y Enfermedades Cardiovasculares, Centro Biomédico, Instituto de Biología, Universidade do Estado do Rio de Janeiro, Rio de Janeiro, Brazil

**Keywords:** gene expression, nutrigenomics, omega-3, epigenetics, molecular biology

## Abstract

**Introduction:**

There exists a correlation between obesity and the consumption of an excessive amount of calories, with a particular association between the intake of saturated and trans fats and an elevated body mass index. Omega-3 fatty acids, specifically eicosapentaenoic and docosahexaenoic acids, have been identified as potential preventive nutrients against the cardiometabolic hazards that are commonly associated with obesity. The objective of this comprehensive review was to elucidate the involvement of long-chain polyunsaturated fatty acids, specifically eicosapentaenoic acid and docosahexaenoic acid, in the modulation of gene expression during the progression of obesity.

**Methods:**

The present analysis focused on primary studies that investigated the association between long-chain polyunsaturated fatty acids, gene expression, and obesity in individuals aged 18 to 65 years. Furthermore, a comprehensive search was conducted on many databases until August 2023 to identify English-language scholarly articles utilizing MeSH terms and textual content pertaining to long-chain polyunsaturated fatty acids, gene expression, obesity, and omega-3. The protocol has been registered on PROSPERO under the registration number CRD42022298395. A comprehensive analysis was conducted on a total of nine primary research articles. All research collected and presented quantitative data.

**Results and Discussion:**

The findings of our study indicate that the incorporation of eicosapentaenoic and docosahexaenoic acid may have potential advantages and efficacy in addressing noncommunicable diseases, including obesity. This can be attributed to their anti-inflammatory properties and their ability to regulate genes associated with obesity, such as PPARγ and those within the *ALOX* family.

**Systematic Review Registration:**

https://www.crd.york.ac.uk/prospero/display_record.php?ID=CRD42022298395, CRD42022298395.

## 1. Introduction

Due to its immense effects on health, medical costs, and mortality, obesity is a public health concern. Over 30 % of adults have been impacted by obesity and overweight (1, 2). Obesity is a multifaceted and complex condition that is typically preventable; it is characterized by an excessive accumulation of adipose tissue that poses a health risk due to its association with type 2 diabetes, hypertension, and hyperlipidemia (3, 4).

The multidisciplinary field of study known as genomics was created to understand genomes’ structure, function, evolution, and mapping (5). In this way, nutrigenomics and nutri-epigenomics enable the chemical analysis of food and the investigation of the metabolites created during food oxidation to modify gene expression either directly or indirectly through epigenetic remodeling (6).

Every meal contains thousands of biologically active substances, many of which may benefit our health. Among these are those related to polyunsaturated fatty acids. Within the unsaturated fatty acids, the long-chain polyunsaturated fatty acids can be further split into the omega-3 and omega-6 groups (7), which are particularly significant. Eicosapentaenoic and docosahexaenoic acids, which are mainly found in fish, shellfish, and marine algae, and arachidonic acid, which is regularly found in chicken and eggs, are examples of common long-chain polyunsaturated fatty acids (8).

Consumption of indulgent foods, saturated lipids, and trans fats is associated with a higher body mass index. According to Swinburn et al. (4), excessive caloric intake and poor diet quality have also been linked to obesity. It is now known, however, that omega-3 fatty acids, such as eicosapentaenoic and docosahexaenoic acids, are adequate nutrients that protect against the cardiometabolic dangers associated with obesity (9). As a result, eicosapentaenoic and docosahexaenoic acids inhibit the innate immune response that TLR4 induces in adipose and trophoblast cells to reduce inflammation in obese pregnant women (10). Also, taking extra eicosapentaenoic and docosahexaenoic acid lowers the amount of triacylglycerol in the blood (11–13).

However, genetic variability and variables such as physical activity, drugs, and food pesticide residues, among other factors, produce multiple results (14). For these reasons, understanding the molecular effects of long-chain polyunsaturated fatty acids, e.g., eicosapentaenoic and docosahexaenoic acids, in regulating gene expression during the development of obesity is a promising goal that could strongly impact dietary choices, considering not only food composition but also its nutrigenomic and nutria-epigenomic properties. Thus, the aim of the study was to describe the role of long-chain polyunsaturated fatty acids, eicosapentaenoic, and docosahexaenoic acids, in the regulation of gene expression during the development of obesity in humans between 18 and 65 years old.

## 2. Materials and methods

The goal of this study was to do a systematic review of quantitative studies that look at how long-chain polyunsaturated fatty acids, especially eicosapentaenoic and docosahexaenoic acids, are implied in gene expression during obesity development in people between the ages of 18 and 65. The protocol was assigned the registration number CRD42022298395 in the PROSPERO database. The review has been performed according to PRISMA (15).

### 2.1. Search strategy and selection criteria

#### 2.1.1. Search strategy

By using MeSH terms (such as “long-chain polyunsaturated fatty acids” AND “gene expression” AND “obesity” AND “omega-3”) and text terms associated with gene expression, nutrigenomics, and omega-3 in accordance with the research question, several databases (MEDLINE, EMBASE, Scopus, and Web of Science) were searched up to August 2023 for original articles and primary quantitative studies in English. The searches were a part of larger searches for several reviews examining a variety of health-related factors, including anthropometric factors, nutritional analysis, and physical analysis. The included studies and relevant reviews’ reference lists were also searched.

#### 2.1.2. Identification of relevant studies

Two reviewers screened titles, abstracts, and papers for inclusion. Discussion with another reviewer helped to resolve differences between reviewers’ results.

#### 2.1.3. Types of study and design

The specific inclusion criteria were: 1. primary quantitative studies or mixed methods studies with a quantitative component (using descriptive or inferential statistics methods, with parametric or non-parametric methods): cross-sectional studies or randomized controlled trials, which report the type of long-chain polyunsaturated fatty acids (eicosapentaenoic and docosahexaenoic acids), measurements of lean or fat-free mass, total body weight, body mass index and 2. studies in English. Research studies were excluded if they: 1. systematic reviews; 2. conference abstracts; 3. editor letters; 4. were not an original investigation published in full; 5. did not provide or specify numerical data; 6. studies realized just in postmenopausal women; 7. studies focused just in population older than 65 years old and 8. studies not focused on the role of long-chain polyunsaturated fatty acids in the regulation of gene expression during the development of obesity, or that do not describe anthropometric measurements, nutritional or physical analyzes.

#### 2.1.4. Population

Men or women who lived in the community, between 18 and 65 years old, and healthy volunteers free of problems that could lead to subsequent illness, such as osteoarthritis, diabetes, insulin resistance, high blood pressure, or high cholesterol. Additionally, exclusion criteria included being pregnant, nursing, or postmenopausal (due to the effects of estrogen fluctuation on endothelial function and other parameters), having an immune system defect, having a current clinical disease (specifically diabetes and gastrointestinal, liver, kidney, stroke, mental, coronary heart, and thyroid disease), consuming medications that interact with serum lipid profiles and weight loss, taking anticoagulant and beta-blocking medications, and taking anti-HIV drugs.

#### 2.1.5. Quality assessment/risk of bias

One reviewer evaluated the approach’s quality using the National Institute for Health and Care Excellence methodology for quantitative studies (16), while a second reviewer verified its accuracy. Discussions between reviewers helped to settle their differences. As a result, no studies were disqualified due to poor quality.

#### 2.1.6. Data synthesis and extraction

Data relating to population and study characteristics of the included studies were extracted by one reviewer and checked by another reviewer (Table 1).

**Table 1:** Characteristics of included studies.

References	Country	Population, setting	Inter details	Investigated outcomes	Study aims	Main results
(17)	CA	N = 744 Cree adults (men between 38.5 and 42.0 years old; and women between 37.6 and 40.6 years old).	Associations between RBC LC n-3PUFA and proinflammatory markers (hs-CRP, IL-6 and TNF-α) were assessed by using multivariate general linear models with adjustment for sex, age, and waist circumference. An arbitrary inflammation score was defined based on the sum of the quartiles of hs-CRP, IL-6 and TNF-α concentrations.	Measurement of RBC fatty acid, hs-CRP, IL-6, TNF-α, total cholesterol, LDL-C, HDL-C, triacylglycerol, fasting insulin, fasting glucose, and toxic metals (that is, lead, mercury, cadmium).	To determine the prevalence of elevated high-sensitivity (hs)-CRP concentrations among the James Bay Cree population from the province of Quebec, Canada. To assess the association between RBC LC n-3PUFA, used as a biological marker of LC n-3PUFA dietary intake, and inflammatory biomarker concentrations.	*Results in men:* Weight (kg): 97.0 (94.9–99.1); BMI (kg/m^2^): 31.8 (31.1–32.4); %BF: 32.9 (31.9–33.9); waist circumference (cm): 109.9 (108.2–111.5)*. Inflammatory markers:* hs-CRP (mg/l): 2.0 (1.8–2.2); IL-6 (pg/ml): 2.1 (2.0–2.3); TNF-α (pg/ml): 2.3 (2.1–2.5) *Results in women:* Weight (kg): 90.1 (88.2–92.0); BMI (kg/m^2^): 34.3 (33.7–35.0); %BF: 44.6 (43.9–45.2); waist circumference (cm): 110.5 (109.0–112.0). *Inflammatory markers:* hs-CRP (mg/l): 2.9 (2.7–3.2); IL-6 (pg/ml): 2.7 (2.5–2.8); TNF-α (pg/ml): 2.6 (2.4–2.8) *EPA (%):* Q1: 0.32. hs-CRP (mg/l): 2.25 (1.97–2.56); IL-6 (pg/ml): 2.51 (2.31–2.73); TNF-α (pg/ml): 2.46 (2.18–2.79) *EPA (%):* Q2: 0.41. hs-CRP (mg/l): 2.53 (2.24–2.86); IL-6 (pg/ml): 2.42 (2.23–2.61); TNF-α (pg/ml): 2.33 (2.07–2.61) *EPA (%):* Q3: 0.52. hs-CRP (mg/l): 2.54 (2.25–2.87); IL-6 (pg/ml): 2.22 (2.05–2.40); TNF-α (pg/ml): 2.55 (2.27–2.86) *EPA (%):* Q4: 0.78. hs-CRP (mg/l): 2.30 (2.00–2.65); IL-6 (pg/ml): 2.35 (2.14–2.57); TNF-α (pg/ml): 2.37 (2.07–2.71) *DHA (%):* Q1: 2.54. hs-CRP (mg/l): 2.17 (1.91–2.48); IL-6 (pg/ml): 2.36 (2.17–2.57); TNF-α (pg/ml): 2.37 (2.23–2.87) *DHA (%):* Q2: 3.07. hs-CRP (mg/l): 2.41 (2.13–2.73); IL-6 (pg/ml): 2.32 (2.14–2.51); TNF-α (pg/ml): 2.22 (1.97–2.50) *DHA (%):* Q3: 3.63. hs-CRP (mg/l): 2.63 (2.32–2.96); IL-6 (pg/ml): 2.38 (2.20–2.57); TNF-α (pg/ml): 2.67 (2.39–3.00) *DHA (%):* Q4: 4.58. hs-CRP (mg/l): 2.42 (2.09–2.80); IL-6 (pg/ml): 2.44 (2.22–2.68); TNF-α (pg/ml): 2.29 (1.99–2.63)
(18)	US	N = 26 subjects with a BMI between 28 and 33 kg/m^2^.	N = 16 women and 10 men with a BMI (kg/m^2^) between 28 and 33 were randomly assigned to consume a diet rich in n–3 PUFAs (3.5% of energy intake) from both plant and marine sources or a control diet (0.5% of energy intake from n–3 PUFAs). For the first 2 week, these diets were consumed under isocaloric conditions; then followed a 12-week period of *ad libitum* consumption that was associated with a moderate loss of body weight in both groups.	Body weight, body fat mass, Plasma total adiponectin and HMW adiponectin	To investigate whether a diet rich in n–3 PUFAs increased plasma concentrations of total or HMW adiponectin in healthy overweight-to-moderately obese men and women.	*CRC1: Control:* BW (kg): 84.9 ± 9.3; FM (kg): 31.5 ± 4.8; Plasma total adiponectin (μg/mL): 4.33 ± 1.9; HMW adiponectin (μg/mL): 1.89 ± 1.33. *n–3 PUFAs:* BW (kg): 87.3 ± 12.9; FM (kg): 33.9 ± 5.6; Plasma total adiponectin (μg/mL): 4.21 ± 2.02; HMW adiponectin (μg/mL): 1.85 ± 1.24 *CRC2: Control:* BW (kg): 84.5 ± 9.1; Plasma total adiponectin (μg/mL): 4.06 ± 1.53; HMW adiponectin (μg/mL): 1.92 ± 1.36*. n–3 PUFAs:* BW (kg): 86.7 ± 12.7; Plasma total adiponectin (μg/mL): 4.35 ± 1.98; HMW adiponectin (μg/mL): 2.08 ± 1.24 *CRC3: Control:* BW (kg): 81.4 ± 9.1; FM (kg): 28.9 ± 6.5; Plasma total adiponectin (μg/mL): 4.48 ± 1.55; HMW adiponectin (μg/mL): 2.01 ± 1.38. *n–3 PUFAs:* BW (kg): 84.5 ± 13.7; FM (kg): 32.0 ± 4.9; Plasma total adiponectin (μg/mL): 4.48 ± 1.88; HMW adiponectin (μg/mL): 2.16 ± 1.27
(19)	CA	N = 254 subjects between 18 and 50 years old, and BMI between 25 and 40 kg/m^2^.	The subjects consumed 3 g/day of n-3 PUFA for 6 weeks. Plasma lipids were measured before and after the supplementation period. Five SNPs in *PLA2G2A*, six in *PLA2G2C*, eight in *PLA2G2D*, six in *PLA2G2F*, 22 in *PLA2G4A*, five in *PLA2G6*, and nine in *PLA2G7* were genotyped.	Anthropometric measurements, biochemical parameters, and SNP genotyping.	To examine whether genetic variations in PLA_2_ genes influence plasma TG levels of healthy overweight adults following an n-3 PUFA supplementation.	*Biochemical analysis:* TG levels decreased in 71.2% of subjects. However, TG increased in 28.8% of them. *Genotyping:* Two SNPs, one from *PLA2G2C* (rs2301475) and one from *PLA2G4A* (rs1569480) were associated with plasma TG levels. Interaction effects between n-3 PUFA supplementation and genotype were observed in one SNP of *PLA2G7* (rs1805018) and four of *PLA2G4A* (rs10752979, rs10737277, rs7540602 and rs3820185).
(20)	US	N = 11 overweight and obese (BMI > 27 kg/m^2^), non- smoking, sedentary, weight stable adult subjects were recruited.	Each subject completed three meal trials: MFA, SFA and O3FA, in a randomized, cross-over design with at least one week between trials first thing in the morning. Subjects were instructed to follow the same pattern of eating for the 3 days prior to each test day. Blood was collected from overnight fasted subjects prior to each test meal (time 0) as well as 1, 2, 4, and 6 h after meal consumption via repeated venipuncture.	Inflammatory (ICAM-1, VCAM-1, TNF-α, CRP), oxidative stress (8-epi y NF-κB), and metabolic (glucose, insulin, non-esterified free fatty acids, and TG) parameters.	To clarify the role of different sources of fat in a high fat meal on inflammation and oxidative stress in overweight and obese adults.	*O3FA group:* CRP (mg/L): postprandial concentrations were higher than 0 h; *TNF-α* (pg/mL): concentrations were lower after meals; *VCAM-*1 (ng/mL): concentrations were lower after meals; *ICAM-1* (ng/mL): no significant changes were found; *8-epi PG_F2α_* (pg/mL): postprandial concentrations were lower than 0 h. *Glucose* (mmol/L): 6.2 ± 0.5 after 6 h postprandial. *Insulin* (mU/L): 11.5 ± 1.9 after 6 h postprandial. *Triglycerides* (mmol/L): 2.28 ± 0.59 after 6 h postprandial. *Non-esterified fatty acids* (mmol/L): 0.39 ± 0.05 after 6 h postprandial. *SFA group:* CRP (mg/L): postprandial concentrations were higher than 0 h; *TNF-α* (pg/mL): concentrations were lower after meals; *VCAM-1* (ng/mL): concentrations were lower after meals; *ICAM-1* (ng/mL): no significant changes were found; *8-epi PG_F2α_* (pg/mL): postprandial concentrations were lower than 0 h. *Glucose* (mmol/L): 5.9 ± 0.4 after 6 h postprandial. *Insulin* (mU/L): 9.8 ± 2.3 after 6 h postprandial. *Triglycerides* (mmol/L): 1.65 ± 0.30 after 6 h postprandial. *Non-esterified fatty acids* (mmol/L): 0.39 ± 0.05 after 6 h postprandial. *MFA group:* CRP (mg/L): postprandial concentrations were higher than 0 h; *TNF-α* (pg/mL): concentrations were lower after meals; *VCAM-1* (ng/mL): concentrations were lower after meals; *ICAM-1* (ng/mL): concentrations were lower after meals; *8-epi PG_F2α_* (pg/mL): no significant changes were found*. Glucose* (mmol/L): 5.9 ± 0.2 after 6 h postprandial. *Insulin* (mU/L): 14.7 ± 4.3 after 6 h postprandial. *Triglycerides* (mmol/L): 2.79 ± 0.74 after 6 h postprandial. *Non-esterified fatty acids* (mmol/L): 0.32 ± 0.04 after 6 h postprandial.
(21)	US	The FFAME Study recruited healthy volunteers (N = 80) to a University of Pennsylvania Clinical and Translational Research Center protocol.	Subjects were randomized to supplementation with n-3 PUFA or placebo and completed an endotoxin challenge (LPS 0.6 ng/kg) after 6–8 weeks treatment. Treatment with “high”-dose n-3 PUFA (3,600 mg/day EPA/DHA) led to a significant reduction in the febrile response to LPS, and a trend toward decreased cytokine response.	Gene expression.	To clarify the role of different sources of fat in a high fat meal on inflammation and oxidative stress in overweight and obese adults.	*Placebo group: Down-regulated genes: CCL18*, *CCL3*, *RGS2*, *SERPINA1*, *APLN*, *FCGR3A*, *FCN1*, *HES1*, *IL1RN*, *IL7R*, *IL8*, *LCP1*, *TREM1*, *FCGR3B*, *IER5L* and *PTGDS*. *Up-regulated genes: FADS1* and *PPARG*. *n-3 PUFA group: Up-regulated genes: CCL18*, *CCL3*, *RGS2*, *SERPINA1*, *APLN*, *FCGR3A*, *FCN1*, *HES1*, *IL1RN*, *IL7R*, *IL8*, *LCP1*, *TREM1*, *FCGR3B*, *IER5L* and *PTGDS*. *Down-regulated genes: FADS1* and *PPARG*. An attenuation of LPS-induced inflammation after n-3 PUFA supplementation was found.
(22)	UK	Healthy normal weight individuals (BMI: 18.5 to 25 kg/m^2^) and healthy individuals living with obesity (BMI: 30 to 40 kg/m^2^, waist circumference ≥ 94 cm males and ≥ 80 cm females) aged 18–65 years.	Fasted blood and an abdominal scWAT biopsy (~1 g) were collected at baseline (week-0) and following a 12-week intervention (week-12) during which participants were randomized to consume either 3 g of fish oil (1.1 g EPA + 0.8 g DHA) or 3 g of corn oil (1.65 g linoleic acid and 0.81 g oleic acid) per day.	Blood analyses, anthropometry, fatty acid composition, endocannabinoid analysis, gene expression and enzyme activity.	To investigate whether intervention with LC n-3PUFA could modify the endocannabinoid system in WAT which may have potential to slow or even reverse the onset of obesity-associated inflammation in the tissue	The predominant FA found in scWAT were oleic acid (18:1n-9), palmitic acid (16:0) and linoleic acid (18:2n-6). People with obesity had more n-6 PUFAs, dihomo-gamma-linolenic acid (20:3n-6) and arachidonic acid (AA; 20:4n-6); as well as n-3 PUFAs, EPA, and DPA. In response to 12-week fish oil intervention, the proportions of scWAT EPA, DPA and DHA significantly increased (by 59, 29 and 36% respectively) in normal weight individuals (*p* = 0.006, <0.001 and < 0.001 respectively) and the proportion of EPA significantly increased (by 56%) in individuals living with metabolically healthy obesity (*p* < 0.001). The proportions of DPA and DHA also increased in individuals living with metabolically healthy obesity (by 9 and 17%) but this did not reach statistical significance. The absolute concentrations of the EPA and DHA containing endocannabinoids EPEA, and DHEA were significantly increased in the scWAT of normal weight individuals in response to 12-week fish oil intervention (*p* = 0.006 and 0.039 respectively). People with obesity had lower proportions of the SFAs, myristic acid (14:0), stearic acid (18:0), arachidic acid (20:0), and the n-3 PUFAs alpha-linolenic acid (18:3n-3) and eicosatetraenoic acid (20:4n-3) in comparison with scWAT from normal-weight individuals. There were no significant changes in the expression of scWAT genes involved in fatty acid metabolite synthesis or degradation in either normal weight individuals or individuals living with metabolically healthy obesity in response to 12-week fish oil intervention.
(23)	UK	Healthy normal weight individuals (BMI: 18.5 to 25 kg/m^2^) and 50 individuals living with obesity (BMI: 30 to 40 kg/m^2^, waist circumference ≥ 94 cm males and ≥ 80 cm females) aged 18–65 years.	Fasted blood and an abdominal scWAT biopsy (~1 g) were collected at study entry (week-0) and following 12 weeks intervention (week-12) during which participants were randomized to consume either 3 g of a fish oil concentrate (providing 1.1 g EPA + 0.8 g DHA) or 3 g of corn oil (providing 1.65 g linoleic acid and 0.81 g oleic acid per day).	Anthropometry, fatty acid composition, oxylipin analysis, gene expression and COX-2 activity.	To investigate obesity associated scWAT inflammation, to identify potential mechanisms by which this occurs in humans, and to assess responses to LC n-3PUFA intervention.	In response to 12-week fish oil intervention, 51 genes were differentially expressed in scWAT in normal weight individuals and 21 genes were differentially expressed in individuals living with obesity. *Normal weight individuals:* 17-HDHA positively correlated with the expression of *ALOX15*, and the proportion of 11-HDHA positively correlated with *ALOX15* and *CYCP1B1* expression. In response to 12-week fish oil intervention, 51 genes were differentially expressed in scWAT in normal weight individuals (*p* < 0.05). The proportions of the arachidonic acid metabolites such as 20-COOH-AA, 14-15-DHET, and AEA were significantly decreased in the scWAT of normal-weight individuals receiving fish oil, but no generation of LC n-3 PUFA metabolites. *Individuals living with obesity:* Obese people exhibit elevated levels of TG, total cholesterol, LDL-C, glucose, and insulin as compared to individuals with a normal weight. People with obesity had more n-6 PUFAs, dihomo-gamma-linolenic acid (20:3n-6) and arachidonic acid (AA; 20:4n-6); as well as n-3 PUFAs, EPA (20:5n-3) and DPA (22:5n- 3); as well as lower levels of n-3 PUFAs, alpha-linolenic acid (18:3n-3) and eicosatetraenoic acid (20:4n-3). 13- HODE was positively correlated with the expression of *PTGS2*, and *PGD3* was positively correlated with *PTGS1* and negatively correlated with *PTGS2*. LXA5, 15-HEPE, and RvE3 were negatively correlated with *ALOX15* expression. Changes in the proportions of LC n-3PUFAs were negatively correlated with markers of insulin resistance. The proportion of EPA was positively correlated with adipose-IR (r = 0.248, *p* = 0.043) and the proportion of DPA with HOMA2-IR and adipose-IR. Saturated and monounsaturated FAs were not altered with either fish oil or corn oil intervention in either group of individuals. In relation to oxylipin metabolism, the expression of the gene encoding *PTGS2* significantly increased by 2.7- fold in scWAT from individuals living with obesity in response to 12-week fish oil intervention.
(24)	US	Participants were between the ages of 18 and 65, non-smokers, with a BMI ≥ 30 kg/m^2^ and no significant weight loss for six months before the study enrollment.	The participants were instructed to consume 500 mg of Nature Made Burp-less Fish Oil capsules. The participants consumed four capsules in the morning alongside their breakfast and an additional four capsules in the evening alongside their meal.	Gene expression, microarray data expression and analysis, plasma and serum analysis, and plasma free fatty acids species profiling.	The objective of this study is to assess the impact of a 3-month daily intake of 4 grams of ω-3PUFA on insulin sensitivity in individuals with obesity who have previously been diagnosed with insulin resistance and systemic inflammation.	A significant reduction in pro-inflammatory macrophage markers, including iNOS (*p* < 0.05), CD68 (p < 0.05), and CD163 (*p* < 0.05) was found in the subcutaneous AT of individuals with obesity and IR. The Inflammatory Response pathway (*S1PR3, TNFAIP6, TNFRSF11A, CHI3L1, SPP1*), the Collagen Catabolic Process route (*MMP9, MMP7, COL8A2*), and the Extracellular Matrix Disassembly pathway (*MMP9, MMP7, SPP1*) were the top four gene pathways found in the pathway enrichment analysis of GOTERMs.
(25)	UK	N = 50 healthy weight individuals (BMI 18.5 to 25 kg/m2) and N = 50 obese individuals (BMI: 30 to 40 kg/m^2^, waist circumference 94 cm for males and 80 cm for females). The participants ranged in age from 18 to 65.	At baseline (week-0) and after a 12-week intervention (week-12), participants were randomly assigned to consume either 3 g of a concentrated fish oil (providing 1.1 g EPA and 0.8 g DHA) or 3 g of corn oil (providing 1.65 g linoleic acid and 0.81 g oleic acid) per day.	Anthropometry, blood analyses, fatty acid composition, gene expression and histochemical analyses.	To describe obesity-related scWAT growth and remodeling, responses to chronic LC n-3 PUFA intervention, and putative mechanisms underlying these findings.	The top upregulated pathways include cytokine signaling, immune cell signaling and differentiation, and activation of inflammatory pathways such as the inflammasome pathway (*p* ≤ 0.05). The enriched pathways involved in tissue remodeling include upregulation of hepatic fibrosis signaling, HIF-1a and VEGF signaling, actin cytoskeleton signaling and dendritic cell maturation, Wnt/β-catenin signaling, and downregulation of inhibition of MMPs (*p* ≤ 0.05). Histochemical staining of scWAT revealed that obese people exhibit tissue hypertrophy where the average adipocyte size was larger, in addition to a greater number of large, very large, and extra-large adipocytes in comparison to normal weight people (*p* ≤ 0.050). There was a greater number of macrophages accumulating in crown like structures (CLS), defined as three or more macrophages aggregating around a single adipocyte, in the scWAT of obese people in comparison to normal weight individuals. The number of CLS per 100 cm^2^ of scWAT was positively correlated with circulating IL-6 (*p* = 0.028) and negatively correlated with circulating adiponectin concentrations (*p* = 0.028). 12-week EPA + DHA significantly modulated the expression of several genes involved in tissue remodeling and expansion processes. These genes are associated with the upregulation of blood vessel remodeling, actin filament binding, cell differentiation, and apoptotic cell clearance in normal weight individuals (*FAM101A*, *FOXC2*, *POF1B*, *KIAA1644*, *FBXO40*, and *TMG2*), and with anatomical structure morphogenesis and the negative regulation of cell proliferation in individuals living with obesity (*MAB21L1*). LC n-3 PUFAs downregulated genes associated with angiogenesis, inflammatory response and circadian rhythm in normal weight individuals, and downregulated genes associated with cell differentiation, negative regulation of cell adhesion, and Wnt signaling in individuals living with obesity (*PROK2, TDRD12*, and *DACT2*).

14-15-DHET: 14,15-dihydroxyicosatrienoic acid; 20-COOH-AA: 20-COOH-arachidonic acid; %BF: body fat percentage; AEA: arachidonoyl ethanolamide; AT: adipose tissue; BMI: body mass index; BW: body weight; CLS: crown like structures; CRC1: After the lead-in period; CRC2: After the isocaloric period; CRC3: After the ad libitum period; CRP: C-reactive protein; RBC: red blood cell; DHA: docosahexaenoic acid; DHEA: docosahexaenoyl ethanolamide; DPA: docosapentaenoic acid; EPA: eicosapentaenoic acid; EPEA: eicosapentaenoyl ethanolamide; FA: fatty acids; FFAME: fenofibrate and omega-3 fatty acid modulation of endotoxemia; FM: fat mass; HDL-C: high-density lipoprotein cholesterol; HMW: high-molecular-weight; hs-CRP: high-sensitivity C-reactive protein; ICAM-1: intercellular adhesion molecule-1; IL-6: interleukin-6; IR: insulin resistance; LA: linoleic acid; LC n-3PUFA: long-chain n-3 polyunsaturated fatty acids; LDL-C: low-density lipoprotein cholesterol; MFA: refined olive oil; n–3 PUFAs: n-3 polyunsaturated fatty acid; n-6 PUFA: n-6 polyunsaturated fatty acid; O3FA: refined olive oil plus 4 g of n-3FA from 8 g fish oil supplement pills; scWAT: subcutaneous white adipose tissue; SFA: refined palm oil; SFAs: saturated fatty acids; TG: triglycerides; TNF-α: tumor necrosis factor-alpha; VCAM-1: vascular cell adhesion molecule-1; WAT: white adipose tissue.

Two researchers systematically analyzed the whole text, meticulously examining the data pertaining to long-chain polyunsaturated fatty acids, gene expression, and obesity. Their objective was to identify relevant information concerning the variables associated with the influence of long-chain polyunsaturated fatty acids on the regulation of gene expression during the developmental stages of obesity in individuals aged 18 to 65 years. The content underwent a thorough examination and was subsequently reorganized into distinct subjects, as presented in Table 2. These were included if the study’s authors built their interpretation and concepts from the initial data.

**Table 2:** Variables involved in role of long-chain polyunsaturated fatty acids in the regulation of gene expression during the development of obesity.

Measurement	Metabolic changes	Evaluation method	References
Anthropometric measurements	Body weight and body fat mass decreased after 12-weeks of n-3 PUFA supplementation.	DXA	(20)
	Higher values in physical measurements of BMI, %BF, body fat mass, waist circumference, and hip circumference was found in people living with obesity.		(23)
Gene expression	SNPs lowered plasma TG levels after n-3 PUFA supplementation	RT-PCR	(19)
	There was attenuation of LPS-induced gene expression with n-3 PUFA supplementation.	RT-PCR	(21)
	scWAT gene expression of *SLC27A2*, *CNR1*, *DAGLA*, *MGLL*, *FAAH*, *SLC27A1* and *SLC27A2* were lower in individuals living with healthy obesity.	RT-PCR	(22)
	A higher expression of *PLA2G2D*, *PLA2G4A* and *PLA2G7* were found in scWAT of individuals living with healthy obesity.	RT-PCR	(22)
	In normal weight individuals, the proportion of 17-HDHA positively correlated with the expression of *ALOX15*, and the proportion of 11-HDHA positively correlated with *ALOX15* and *CYCP1B1* expression.	RT-PCR	(23)
	In obese people, 13-HODE was positively correlated with the expression of *PTGS2*, and *PGD3* was positively correlated with *PTGS1* and negatively correlated with *PTGS2*.	RT-PCR	(23)
	The mRNA expression of *CYP1B1, ALOX5* and *PTGS1* were upregulated in individuals with obesity.	RT-PCR	(23)
	*ALOX12* mRNA expression is negatively correlated with %BF.	RT-PCR	(23)
	*ALOX5* mRNA expression is positively correlated %BF.	RT-PCR	(23)
	The mRNA expression of *ARG-1, CD68, CD163, ADIPOQ,* and *LEP* was downregulated following the administration of n-3 PUFA supplementation.	RT-PCR	(24)
	The top ten upregulated genes include *EGFL6*, *MMP7*, *MMP9*, *DCSTAMP*, *SPP1*, *COL11A1*, *COMP*, *TNC*, *COL4A2*-*AS2*, and *LAMC3* (*p* < 0.005). Top downregulated genes included *COL9A3*, *COL6A6*, and *AZGP1* (*p* ≤ 0.003).	RT-PCR	(25)
	Higher expression of *ANGPT2*, *HIF-1a*, *EGFL6*, several *MMP* genes, and *GDF15* was observed in individuals living with obesity (*p* ≤ 0.008).	RT-PCR	(25)
Inflammation	Individuals with obesity had reduced levels of various compounds, including 9-HpODE; 9-oxo-ODE; 13-oxo-ODE; 12,13-DiHOME; 20-COOH-AA; 11,12-DHET; LTD4; LXB4; HXA3; 9-HOTrE; RvE1; 8-HDHA; 14-HDHA; 15-HDHA; 17-HDHA; 20-HDHA; and RvD2. Additionally, decreased quantities of 4-HDHA and 11-HDHA were observed in these individuals.	UPLC-MS	(23)
	A significant reduction was observed in the plasma concentrations of MCP-1, INF-γ, IL-2, IL-8, IL-10, IL-4, IL-1B, IL-12, TNF-α, and GM-CSF after three months of FO supplementation.	ELISA	(24)
	There were lower proportions of oxylipins derived from n-3 and n-6 PUFA in individuals living with obesity.	RT-PCR	(25)
Biomarkers	Levels of hs-CRP, TNF-alpha and inflammation score are inversely associated with n-3 DPA levels of RBC in Cree adults.	Nephelometry/ELISA	(17)
	PUFA n-3 ingestion decreases ICAM-1 levels.	ELISA	(20)
	At the end of the 12-weeks fish oil intervention, EPA and DHA were higher in scWAT.	RT-PCR	(22)
	Changes in scWAT DPA and DHA were negatively correlated with markers of insulin resistance.	ELISA	(23)
Adiponectin	n-3 PUFA supplementation increases plasma concentrations of total adiponectin in overweight or moderately obese subjects.	ELISA	(20)
	After n-3 PUFA supplementation, plasma levels and adipose tissue mRNA levels of adiponectin were significantly increased (p < 0.05)	ELISA/RT-PCR	(24)

11,12-DHET: 11,12-dihydroxy-eicosatrienoic acid; 12,13-DiHOME: 12,13-dihydroxy-octadecenoic acid; 13-oxo-ODE: 13-oxo-octadecadienoic acid; 20-COOH-AA: 20-COOH-arachidonic acid; 9-HOTrE: 9-hydroxy-octadecatrienoic acid; 9-HpODE: 9-hydroperoxy-octadecadienoic acid; 9-oxo-ODE: 9-oxo-octadecadienoic acid; %BF: percentage of body fat; ANGPT2: angiopoietin-2; AZGP1: alpha-2 glycoprotein-1; DACT2: disheveled binding antagonist of beta catenin-2; DHA: docosahexaenoic acid; DPA: docosapentaenoic acid; DXA: dual-energy x-ray absorptiometry; EGFL6: epidermal growth factors like protein-6; ELISA: enzyme-linked immunosorbent assay; EPA: eicosapentaenoic acid; FAM101A: refilin-A; FBXO40: f box protein-40; FO: fish oil; FOXC2: forkhead box-C2; GDF15: growth/differentiation factor-15; HXA3: hepoxilin A3; hs-CRP: high-sensitivity C-reactive protein; ICAM-1: intercellular adhesion molecule 1; KIAA1644: shisha like-1; LPS: lipopolysaccharide; LTD4: u-leukotriene D4; LXB4: lipoxin B4; MAB21L1: MAB-21 like-1; mRNA: messenger RNA; n-3 DPA: n-3 docosapentaenoic acid; n-3 PUFA: n-3 polyunsaturated fatty acid; n-6 PUFA: n-6 polyunsaturated fatty acid; POF1B: actin binding protein; PROK2: prokineticin-2; RT-PCR: real-time PCR; RBC: red blood cells; RvD2: resolving D2; RvE1: resolvin E1; scWAT: subcutaneous white adipose tissue; SNPs: single nucleotide polymorphisms; TDRD12: tudor domain containing-12; TG: triglycerides; TMG2: transglutamase-2; UPLC-MS: Ultra-performance liquid chromatography-mass spectrometry.

## 3. Results

**Figure 1:**
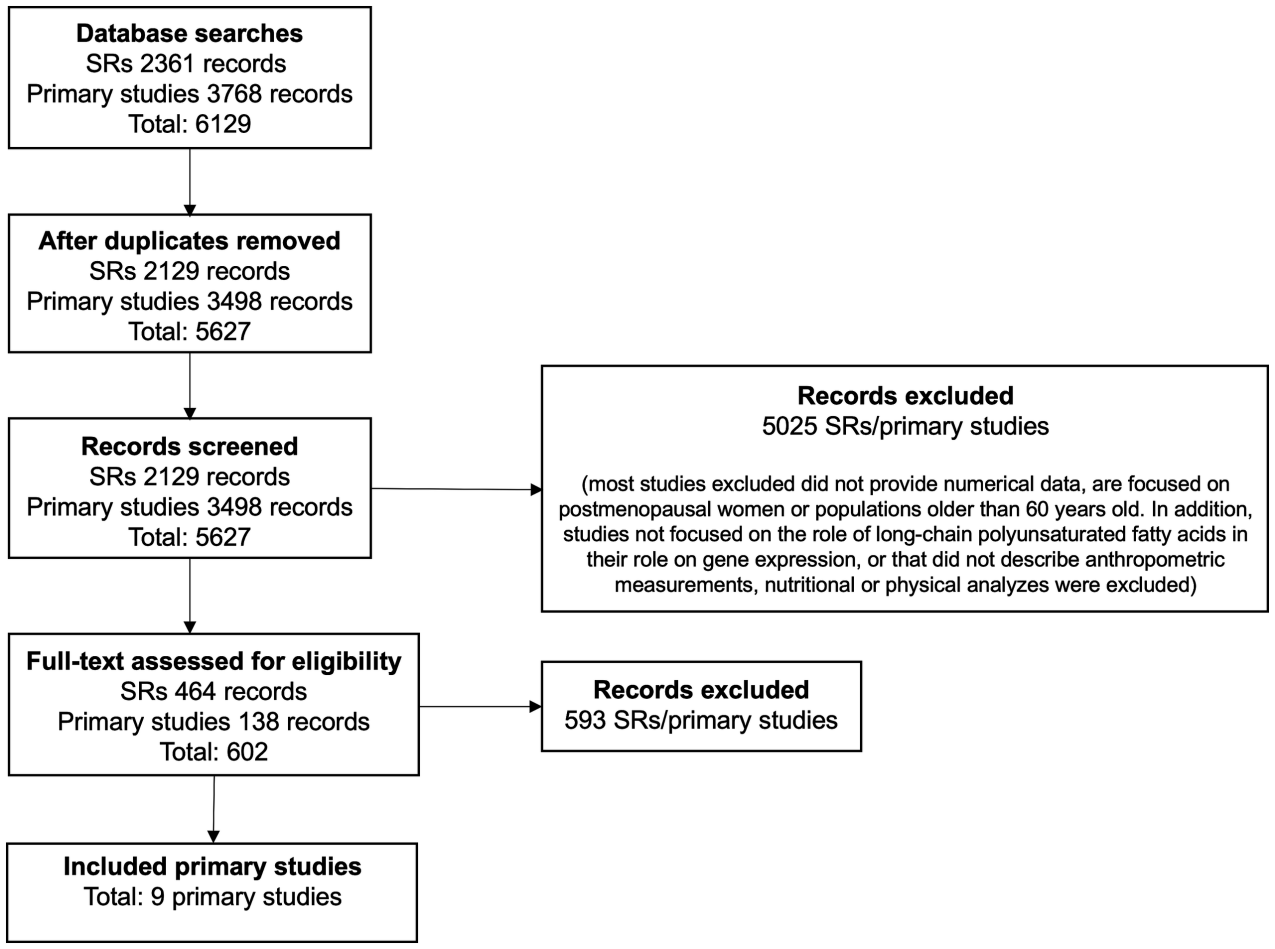
PRISMA flow diagram.

Figure 1 illustrates the flow chart for the study selection process from the nine papers identified (17–25). A summary of the included studies and the populations, settings, and contexts in which they were conducted are shown in Table 1.

### 3.1. Description of included studies

Four publications from the primary studies were realized in the US, two in Canada, and three in the United Kingdom. Nine papers in all were examined. The papers collected and reported quantitative data through clinical trials or experimental studies (Table 1).

Participants from both sexes were included in every study (17–25). Additionally, seven investigations on white people (18, 20–25), one on the Cree population (17), and one on residents of Quebec City were carried out (19).

Details of the long-chain polyunsaturated fatty acids investigated in each study are shown in Table 1. Only omega-3 long-chain polyunsaturated fatty acid studies have been included (17–25).

### 3.2. Quality assessment

Table 3 displays the quality assessment outcomes and evaluation standards for thorough research. Studies’ overall internal and external validity quality was often high or moderate. No studies were removed due to poor quality.

**Table 3:** National Institute for health and care excellence methodology checklist: quantitative studies.

References	Study design	Population			Method of allocation to intervention (or comparison)										Outcomes						Analyses						Summary	
		1	2	3	4	5	6	7	8	9	10	11	12	13	14	15	16	17	18	19	20	21	22	23	24	25	26	27
(17)	Cross-sectional study	+	+	−	+	++	NR	NA	NA	NA	++	+	NA	NA	++	+	++	++	+	++	++	++	+	NR	+	++	+	+
(18)	Randomized controlled trial	−	−	+	++	++	++	+	+	+	++	+	++	++	−	++	++	+	++	+	+	+	+	+	+	+	+	+
(19)	Cross-sectional study	+	+	+	++	++	++	+	++	++	++	++	++	++	+	+	++	++	NA	+	++	++	+	+	+	++	+	+
(20)	Randomized controlled trial	+	−	−	++	++	++	++	++	++	++	++	++	+	++	++	++	++	++	−	++	++	+	+	++	++	++	+
(21)	Randomized controlled trial	+	+	+	++	++	++	++	++	++	++	++	++	++	++	++	++	++	++	+	++	++	−	++	++	++	++	+
(22)	Randomized controlled trial	++	+	++	++	++	++	++	++	++	++	+	NR	NR	++	++	++	++	++	++	++	++	−	NR	+	++	++	++
(23)	Randomized controlled trial	++	+	++	++	++	++	++	+	++	++	+	NR	NR	++	++	+	++	++	++	++	++	+	NR	++	++	++	++
(24)	Randomized controlled trial	++	−	+	++	++	+	NR	++	NA	++	++	NR	NR	++	++	++	++	++	++	++	NR	+	NR	++	+	++	++
(25)	Randomized clinical trial	++	−	+	++	++	++	++	++	NA	++	++	++	++	++	++	++	++	++	++	+	NR	+	NR	++	++	++	++

Key to headings: Population 1. Is the source population or source area well described? 2. Is the eligible population or area representative of the source population or area? 3. Do the selected participants or areas represent the eligible population or area? Method of allocation to intervention (or comparison) 4. Allocation to intervention (or comparison). How was selection bias minimized? 5. Were interventions (and comparisons) well described and appropriate? 6. Was the allocation concealed? 7. Were participants or investigators blind to exposure and comparison? 8. Was the exposure to the intervention and comparison adequate? 9. Was contamination acceptably now? 10. Were other interventions similar in both groups? 11. Were all participants accounted for at study conclusion? 12. Did the setting reflect usual UK practice? 13. Did the intervention or control comparison reflect usual UK practices? Outcomes 14. Were outcome measure reliable? 15. Were all outcome measurements complete? 16. Were all important outcomes assessed? 17. Were outcomes relevant? 18. Were there similar follow-up times in exposure and comparison groups? 19. Was follow-up time meaningful? Analyses 20. Were exposure and comparison groups similar at baseline? If not, were these adjusted? 21. Was intention to treat (ITT) analysis conducted? 22. Was the study sufficiently powered to detect an intervention effect (if one exists)? 23. Were the estimates of effect size given or calculable 24. Were the analytical methods appropriate 25. Was the precision of intervention effects given or calculable? Were they meaningful? Summary 26. Are the study results internally valid (i.e., unbiased)? 27. Are the findings generalizable to the source population (i.e., externally valid)? (National Institute for Health and Care Excellence (NICE) Methodology checklist: quantitative studies https://www.nice.org.uk/process/pmg4/chapter/appendix-f-quality-appraisal-checklist-quantitative-intervention-studies). Not applicable (NA): It is reserved for those study design aspects that are not applicable given the study design under review; Not reported (NR): it is reserved for those aspects in which the study under review fails to report how they have (or might have) been considered; −: it is reserved for those aspects of the study design in which significant sources of bias may persist; +: it indicates that either the answer to the checklist question is not clear from the way the study is reported, or that the study may not have addressed all potential sources of bias for that particular aspect of study design and; ++: it indicates that for that particular aspect of study design, the study has been designed or conducted in such a way as to minimize the risk of bias.

### 3.3. Body size and composition

The effects of long-chain polyunsaturated fatty acids consumption on body composition can be addressed using anthropometric measurements such as waist circumference, fat mass, lean mass, and body mass index; however, other analyses related to the effects of long-chain polyunsaturated fatty acids consumption on gene expression through biomarkers, such as cytokines, enzyme activity, fatty acid composition, oxylipins, and biochemical markers, could be used too.

While studies had differing objectives, populations, and contexts, some themes were consistently repeated across several studies (Table 2). The main themes described have been identified in high-quality studies. None were identified in low-quality studies.

### 3.4. Relation between long-chain polyunsaturated fatty acids and obesity

The use of biomarkers or anthropometric measurements to evaluate the effects on body composition has been indicated in almost all studies (Table 2). In effect, Kratz et al. (18). found decreased body weight and body fat after omega-3 long-chain polyunsaturated fatty acid supplementation (*p* < 0.001 and *p* = 0.002, respectively).

### 3.5. Regulation of gene expression

Gene expression refers to the mechanism via which the genetic information contained within a gene is translated into a functional product or activity. Western blot, mRNA analysis, and enzyme-linked immunosorbent assays are commonly employed techniques for quantifying the expression levels of gene products.

Accordingly, *PLA2G2A* and *PLA2G4A* genes were up-regulated by omega-3 polyunsaturated fatty acids supplementation (19, 22), whereas *SLC27A2*, *CNR1*, *DAGLA*, *MGLL*, *FAAH*, *SLC27A1*, and *SLC27A2* genes were found to be down-regulated in people living with healthy obesity (22). Additionally, the *ALOX5* gene shows a negative correlation with body fat and fat mass, while the *ALOX12* gene has a positive correlation with both of them (23).

### 3.6. Biomarkers

Fatty tissue is believed to function as a very sophisticated organ system (26). The primary cause of metabolic disorders is white adiposse tissue, particularly in the abdomen (27–29). In white adiposse tissue, adipocytes release various hormones and inflammatory substances, such as cytokines (24, 30). Leptin, adiponectin, resistin, and visfatin are the hormones typically linked to adipose tissue; nevertheless, adipocytes can also release IL-6 and TNF-α (31, 32).

In this regard, hs-CRP and IL-6 were directly associated with docosahexaenoic acid levels, whereas IL-6 and TNF-α were inversely associated with eicosapentaenoic acid and omega-3 long-chain polyunsaturated fatty acid levels (17). On the other hand, after eating a meal high in omega-3 fatty acids, plasma CRP increased over time, whereas TNF-α and VCAM-1 tended to decline (20). A meal richer in omega-3 fatty acids had a more significant postprandial effect on nuclear factor-κB over the following 4 h than a meal high in saturated fat. However, the cumulative impact of the meals was not statistically significant (20). Similarly, increased docosahexaenoic acid values were discovered following the omega-3 long-chain polyunsaturated fatty acid intervention (22, 23). Two genes that are significant for gene-nutrient expression, have been markedly up-regulated in the omega-3 long-chain polyunsaturated fatty acids supplemented group (21). Results are shown in detail in Tables 1, 2.

## 4. Discussion

This systematic review collates and synthesizes evidence from nine quantitative studies relating long-chain polyunsaturated fatty acid consumption (eicosapentaenoic and docosahexaenoic acids) with the regulation of gene expression and anti-inflammatory effects during the development of obesity in humans between 18 and 65 years old.

### 4.1. Summary of Key findings and interpretation

It is important to note that our searches aimed mainly at the evaluation of gene expression and anti-inflammatory activity after eicosapentaenoic and docosahexaenoic acids intake, because they are poorly investigated compared to other macro and micronutrients.

Long-chain polyunsaturated fatty acids have emerged as a potential protective nutrient against the cardiometabolic risks associated with obesity (33), where a higher body mass index has been linked with low omega-3 status among adults (34–36). In this sense, our study found a decrease in body composition and fat mass after eicosapentaenoic and docosahexaenoic acid intake (18). Indeed, previous studies described a decreased body mass index after consuming eicosapentaenoic acid; where a 4.35% weight loss was found after 12 weeks (37, 38), confirming its protective role.

There exists a widespread association between obesity and inflammation (17, 21, 23, 25, 39). This correlation is characterized by an elevation in mRNA levels of IL-6, IL-12, IFN, and CXCL10 chemokines in individuals with obesity. Adiponectin activity exhibits a protective effect in multiple physiological processes, including energy metabolism, inflammation, and cell proliferation. Furthermore, it has been implicated in the mitigation of chronic non-communicable conditions such as diabetes mellitus (40–42). The present study has observed elevated levels of PPAR-γ and adiponectin subsequent to the intake of eicosapentaenoic and docosahexaenoic acid (18, 23, 25). These findings provide support for the beneficial effects of omega-3 long-chain polyunsaturated fatty acids. A noteworthy discovery pertaining to eicosapentaenoic and docosahexaenoic acids is their ability to mitigate inflammation triggered by lipopolysaccharide (21). This implies that eicosapentaenoic and docosahexaenoic acids serve as primary inhibitors of this inflammatory response (17, 43).

Both eicosapentaenoic and docosahexaenoic acids have the primary effect of lowering triglyceride levels by reducing the synthesis of very-low-density lipoprotein-triglycerides in the liver (44). In addition, women living with obesity were observed to have lower triglyceride levels after consuming eicosapentaenoic and docosahexaenoic acids (45, 46). However, even though consuming omega-3 long-chain polyunsaturated fatty acids led to lower levels of fatty acids (19, 22, 47), no definite effects on triglycerides have been reported (19, 20).

The American Heart Association has described the advantages of omega-3 long-chain polyunsaturated fatty acid consumption to lower hypertriglyceridemia and the variables that affect its measurement to examine these discrepancies (13). In this instance, phospholipase A2 polymorphisms were investigated, and triglyceride levels in humans living with obesity were found to be correlated with SNPs (19). In addition, alterations in the expression of certain enzymes, like PLA2G2D, would reduce eicosapentaenoic and docosahexaenoic acids in murine models (48), which could partially account for the disparity.

Dietary polyunsaturated fatty acid intake may control the parameters related to obesity through different epigenetic mechanisms. There is current information on the genetic modulation of *ALOX5* and *ALOX15* expression during obesity (23), where *ALOX5* promotes leukotrienes, lipoxins, and resolvins production (46). However, *ALOX12* and *ALOX15* genes appear to have anti-inflammatory and inflammatory effects, respectively (49). Likewise, *ALOX12* pro-inflammatory activity was decreased after omega-3 intake (23, 25, 37). Although *ALOX12* and *ALOX15* could have pro-inflammatory activity, our results might be explained because *ALOX12* and *ALOX15* are involved in eicosapentaenoic and docosahexaenoic acid metabolism (49). However, more studies are still required to understand the relationship between genomics, obesity, and polyunsaturated fatty acids.

After polyunsaturated fatty acid consumption, there would be a correlation between obesity and inflammation, decreasing pro-inflammatory genes and cytokines expression, such as VCAM-1, 8-epi-prostaglandin-F2α (8-EPI), and TNF-α (20). According to previous studies, polyunsaturated fatty acids decrease sVCAM-1 and TNF-α in people living with obesity (24, 40, 42, 46).

While inflammation serves a healthy function, uncontrolled inflammation can have negative consequences, leading to tissue damage and the development of many diseases. In these circumstances, inflammation has a self-limiting nature and the initiation of active resolution mechanisms. At the core of these processes lies the production of specialized pro-resolving lipid mediators (SPMs) derived from EPA and DHA. Resolvins, protectins, and maresins, which have been extensively characterized in cellular and animal models, are among the compounds under consideration (50, 51).

SPMs encompass a collection of anti-inflammatory mediators that can be categorized into four distinct families, namely lipoxins (LX), resolvins (Rv), protectins (PD), and marosins (MaR) (52). SPMs are produced from the same precursors as proinflammatory mediators; however, the mechanisms exhibit significant differences. These structures have the ability to selectively attach to particular receptors, thereby reinstating homeostasis through the reduction of cellular activity and inflammation (53). In recent times, there has been an increasing recognition of the potential therapeutic value of mediators in the treatment of inflammatory illnesses. Research on the physiology of resolution has led to the exploration of novel research domains, spanning from fundamental physiology and pharmacology to the identification of potential therapeutic targets (54).

Previous studies have demonstrated the efficacy of SPMs in attenuating the advancement of cardiovascular disease (CVD) through the modulation of many molecular pathways implicated in the pathogenesis of CVD (54–57). SPMs refer to specialized pro-resolving mediators, which are the metabolic byproducts derived from ω-3 and ω-6 polyunsaturated fatty acids (PUFAs) through the enzymatic actions of lipoxygenase (LOX), cyclooxygenase-2 (COX-2), and, to a lesser extent, cytochrome P450.

Animal studies have shown that not having enough lipooxygenase leads to more epoxyeicosatrienoic acid and SPMs being made. These variables have the ability to regulate the activation of proinflammatory pathways, promoting cardiac repair and minimizing cardiac remodeling in both acute and chronic heart failure conditions (58, 59). The cellular impacts of SPMs are derived from their interaction with distinct G protein-coupled transmembrane receptors (GPCRs), namely ALX/FPR2, GPR32/DRV1, ChemR23, BLT1, GPR37, and GPR18/DRV2 (60). In general, SPMs exhibit common signaling pathways such as intracellular phosphorylation cascades and gene regulation, with the exception of PD1, which induces intracellular calcium elevation and subsequently activates calcium-dependent signaling pathways (61).

The sole receptor previously believed to be accountable for the biological consequences of LX is the formyl peptide receptor 2 (FPR2), or ALX. In addition to LXA4, the ALX/FPR2 receptor is also stimulated by RvD1 and RvD3. However, as of now, no receptors that bind to LXB4 have been discovered (62). The influence of RvD1 on the phosphorylation of AKT through the PI3K pathway results in the inhibition of proinflammatory effects mediated by NF-kB. Additionally, it has been observed that it augments ERK1/2 phosphorylation through MEK1/2 activation, hence eliciting anti-inflammatory responses (63, 64). The activation of the nuclear factor erythroid 2-related factor 2 (NRF2) occurs through the interaction with LXA4, leading to the induction of phosphorylation at the Ser40 residue. This phosphorylation event subsequently facilitates the translocation of NRF2 into the nucleus. Phosphorylated NRF2 has the capability to establish a heterodimeric association with sMAF, leading to the formation of a complex that can bind to the antioxidant response element (ARE). This binding event subsequently triggers the transcriptional activation of various antioxidant genes, including HO-1, NQO-1, SOD, and TXN (65).

The RvD1 receptor, known as GPR32/DRV1, belongs to the GPCR family and exhibits a similar binding affinity for RvD3 and RvD5 (25). The connection between Resolvin D1 (RvD1) and G-protein-coupled receptor 32 (GPR32)/DRV1 leads to enhanced production of several microRNAs (miRNAs) in macrophages. One such miRNA is miR-208a, which hinders the transcription process of tumor suppressor protein 4 (PDCD4) and consequently leads to an elevation in the levels of interleukin-10 (IL-10). Furthermore, it has been observed that there is an augmentation in the production of miR146b, a molecule that effectively suppresses the transcription of NF-κB (66). Furthermore, it is worth noting that GPR18/DRV2 belongs to the same family and has been identified as the sole receptor for RvD2. The activation of this entity induces the ERK1/2, PKA, or PLC pathway in order to facilitate resolution. In contrast, GPR37 exclusively interacts with PD1, hence inhibiting PKA and triggering calcium-dependent signaling pathways that ultimately result in the promotion of phagocytosis and the modulation of cytokine production (67).

The expression of ALX/FRP2, GPR32/DRV1, ChemR23, and GPR18/DRV2 is not restricted only to immune system cells. These receptors have also been detected in vascular smooth muscle cells (VSMC), endothelial cells, and atherosclerotic lesions (68–70). Moreover, when endothelial cells were exposed to docosahexaenoic acid (DHA), there was a notable decrease in the adhesion and migration of immune cells. This effect was significantly attenuated when the receptors for Resolvin D1 (RvD1), namely ALX/FPR2 and GPR32/DRV1, were blocked.

However, there are a number of pathogenic and therapeutic factors that can affect the intricate involvement of endogenous SPMs in the context of chronic inflammation and disrupt their typical functioning. For instance, multiple studies conducted on animal models have demonstrated that the resolving response may be altered by the combination of age and obesity generated by a diet rich in PUFAs (71, 72). The observed phenomenon could potentially be attributed to the increased movement of neutrophils and the presence of a significant number of proinflammatory cytokines and lipid mediators within the cardioplegic and cardiorenal networks (71, 72). Molecules such as doxorubicin (73), carprofen (74), and FPR2 inhibitors (75) have the ability to disrupt immunometabolic responses by decreasing the synthesis of SPMs and altering the maturation of leukocytes. Consequently, the resolution of the chronic inflammatory process is prolonged.

### 4.2. Scope and limitations

The goal was to seek an explanation that would reconcile prior conflicting findings concerning the role of long-chain polyunsaturated fatty acids, eicosapentaenoic, and docosahexaenoic, in the regulation of gene expression during the development of obesity. Our review confirms that eicosapentaenoic acid and docosahexaenoic acid participate in the regulation of gene expression by modifying components of fatty acid metabolism. Indeed, eicosapentaenoic acid and docosahexaenoic acid generate epigenetic changes in fatty acid metabolism, which are evidenced through changes in gene expression, anthropometric measurements, biochemical markers, and inflammation.

Unfortunately, our review had some limitations, i.e., a low quantity of articles linking omega-3 polyunsaturated fatty acids to gene expression and their influence on obesity. Also, the intervention times were highly variable between studies, with significant differences in the number of weeks and days. Finally, some studies did not provide sufficient data to compare the results obtained before and after the intervention, and some did not even incorporate the baseline measurements for the parameters studied, which limited the extraction of information.

## 5. Conclusion

Obesity is an alarmingly increasing public health issue. Obesity prevention is a critical factor in controlling obesity-related non-communicable diseases, including diabetes, cardiovascular disease, stroke, hypertension, cancer, and psychological problems.

Our results suggest that eicosapentaenoic and docosahexaenoic acids could be beneficial and effective against noncommunicable diseases, such as obesity, in people over 18 years old thanks to the anti-inflammatory role and the modulation of obesity-regulating genes such as PPARγ and those belonging to the *ALOX* family.

The preclinical evidence suggests that SPMs have potential as innovative and promising agents in the prevention and management of CVD. This can be achieved by either enhancing the endogenous synthesis of SPMs through supplementation of PUFAs and other molecules that promote their production, or by administering exogenous synthetic analogs of SPMs, either alone or in conjunction with other drugs that protect the heart. Moreover, it has been observed in both *in vitro* and *in vivo* preclinical investigations that SPMs enhance host defenses, which distinguishes them from existing anti-inflammatory therapies. Consequently, it is anticipated that these findings will lead to the development of novel guidelines pertaining to the utilization of SPMs in the management of cardiovascular disease.

## Data availability statement

The original contributions presented in the study are included in the article/supplementary material, further inquiries can be directed to the corresponding authors.

## Author contributions

CS: Conceptualization, Writing – original draft, Writing – review & editing. KN: Data curation, Formal analysis, Investigation, Writing – original draft, Writing – review & editing. LM: Data curation, Formal analysis, Investigation, Writing – original draft, Writing – review & editing. BR: Data curation, Formal analysis, Investigation, Writing – original draft, Writing – review & editing. VS-M: Supervision, Writing – original draft, Writing – review & editing. JF: Writing – original draft, Writing – review & editing, Conceptualization.
